# The regulatory effects of water-fertilizer integration on maize canopy uniformity and yield: a comprehensive evaluation based on multi-source UAV data

**DOI:** 10.3389/fpls.2026.1778586

**Published:** 2026-07-01

**Authors:** Yibo Wei, Jiangchuan Fan, Zexi Li, Feifei Shan, Jiangfeng Duan, Mengdi Shi, Xinyi Wang, Qian Zhao

**Affiliations:** 1 Information Technology Research Center, Beijing Academy of Agriculture and Forestry Sciences, Beijing, China; 2 Intelligent Equipment Technology Research Center, Beijing Academy of Agricultural and Forestry Sciences, Beijing, China; 3 Jilin Academy of Agricultural Sciences (Northeast Agricultural Research Center of China), Changchun, China

**Keywords:** canopy uniformity, fertilizer-water integration, high-throughput phenotyping, maize (Zea mays L.), UAV multisource data, yield

## Abstract

**Introduction:**

High and stable maize yields are central to ensuring global food security. However, canopy growth differentiation caused by traditional water and fertilizer management patterns remains a key bottleneck constraining maize productivity improvement. This study aimed to elucidate the regulatory effects of water-fertilizer integration on maize canopy uniformity and yield.

**Methods:**

Forty maize varieties were used as experimental materials, and two treatments were set up for comparison: water-fertilizer integration (WF) and conventional water-fertilizer management (CK). Based on RGB and multispectral data acquired by an unmanned aerial vehicle (UAV), a three-dimensional canopy uniformity evaluation system encompassing structural, coverage, and physiological dimensions was established. Through significant difference analysis, correlation analysis, and comprehensive evaluation, the regulatory mechanisms of water-fertilizer integration and varietal response differences were systematically investigated.

**Results:**

(1) Water-fertilizer integration significantly enhanced three-dimensional canopy uniformity. Structurally, the spatial autocorrelation coefficient (Moran’s I) for plant height increased by 49.9%, while height standard deviation decreased by 50.5%. In terms of coverage, the spatial coefficient of variation for canopy cover decreased by 47.4% and gap rate decreased by 67.3%. Physiologically, the NDVI coefficient of variation decreased by 44.4%, and the standard deviation of estimated chlorophyll content decreased by 55.5%. (2) Canopy uniformity indices showed significant positive correlations with yield. The Pearson correlation coefficient between the comprehensive canopy uniformity index and yield reached 0.88 (P<0.01). Key correlated indicators included the spatial autocorrelation coefficient of canopy height, spatial coefficient of variation of canopy cover, and coefficient of variation of NDVI. (3) Varieties exhibited significant differences in response to water-fertilizer integration. Some varieties showed over 30% improvement in comprehensive uniformity index and 28% yield increase, while a few varieties responded weakly. (4) Water-fertilizer integration enhances yield through a chain mechanism: "uniform water and fertilizer supply → uniform root growth → three-dimensional canopy uniformity → efficient resource utilization → yield increase."

**Discussion:**

The canopy uniformity evaluation system established in this study provides a novel approach for quantifying crop population phenotypes. The identified regulatory mechanisms and variety adaptability offer theoretical support and practical guidance for precision water-fertilizer management and high-yield cultivation models in maize.

## 1 Introduction

Maize (Zea mays L.), as one of the world’s three major food crops, relies on high and stable yields as a core pillar for ensuring national food security and sustainable agricultural development. Under the dual pressures of intensifying climate change, water scarcity, and low fertilizer utilization rates, the traditional water-fertilizer management model—combining flood irrigation with concentrated fertilization—can no longer meet the precision demands of modern maize production. This often leads to uneven spatial distribution of water and nutrients in the field, triggering uneven canopy growth. — resulting in uneven plant heights, noticeable gaps in canopy coverage, and significant heterogeneity in physiological states (growth vigor, nutrients, moisture). This ultimately leads to inefficient light energy capture and imbalanced resource competition, becoming a key bottleneck constraining yield increases. Water-fertilizer integration technology, achieved through drip irrigation systems, enables simultaneous and precise delivery of water and nutrients (Zheng et al., 2016). This provides a crucial technical pathway for establishing efficient crop populations and overcoming the shortcomings of traditional management practices. Consequently, exploring maize water-fertilizer management technologies that balance high yields, efficiency, and environmental friendliness, while elucidating their underlying mechanisms for regulating yield formation, has become an urgent priority in global agricultural research.

Traditional maize water and fertilizer management primarily relies on flood irrigation combined with concentrated fertilization. Under this model, irrigation water is applied via furrow or ditch flooding, resulting in uneven spatial distribution of moisture in the field. Areas near the water inlet suffer from waterlogged, oxygen-deprived soil that impedes root respiration, while distant regions may experience drought stress. Water use efficiency remains below 40%. Fertilizer application primarily involves basal application and 1–2 concentrated topdressings. Granular fertilizers spread over the soil surface and then permeate through irrigation, easily causing localized nutrient accumulation (triggering luxury absorption and leaching losses) or insufficient diffusion (resulting in nutrient deficiencies). Nitrogen fertilizer loss rates reach as high as 40–50% (Li et al., 2021; Yi et al., 2022). This spatiotemporal heterogeneity in water and fertilizer supply directly induces growth differentiation within maize canopies: plants exhibit significant height variation (with a maximum height difference of 97.7 ± 6.2 cm under conventional treatments), noticeable gaps in canopy coverage (gap rates as high as 9.8 ± 1.7%), and significant physiological heterogeneity (growth vigor, nutrients, water status). Ultimately, this results in inefficient light energy capture (photosynthetic blank zones exceeding 15% coverage) and imbalanced resource competition, becoming a key bottleneck constraining yield enhancement (Zamora-Re et al., 2020; Jiang et al., 2024). For instance, studies indicate that under conventional water and fertilizer management, weak plants constitute 15–20% of maize populations, yielding only 40–60% of normal plants and severely hindering the realization of population yield potential (Wu et al., 2019). Furthermore, traditional water and fertilizer management practices impose severe environmental pressures. Fertilizer leaching has led to a year-on-year increase in water eutrophication rates in major Chinese river basins such as the Yangtze and Yellow Rivers. Extensive flood irrigation exacerbates water supply-demand conflicts in arid northern regions, contradicting the goals of green and sustainable agricultural development (Zhang et al., 2023; Hussain et al., 2025).

Water-fertilizer integration technology, as one of the core technologies in precision agriculture, enables the simultaneous and precise delivery of water and nutrients through drip irrigation systems. This provides a crucial technical pathway for overcoming the shortcomings of traditional management practices and establishing highly efficient crop populations (Al-Sammarraie et al., 2021; Bansal et al., 2021). This technology employs a “low-dose, high-frequency” approach to uniformly deliver soluble fertilizers with irrigation water to the crop root zone, ensuring soil water and nutrient conditions remain within optimal ranges (maintaining soil relative moisture above 70% of field capacity and synchronizing nutrient supply with crop demand). This effectively prevents issues such as drought stress and nutrient imbalance (Anjum et al., 2023; S et al., 2023). Existing research indicates that water-fertilizer integration technology can increase maize nitrogen fertilizer utilization by 20–30%, improve irrigation water use efficiency by 35–45%, and boost yield by 10–25% (Fan et al., 2020; Bwambale et al., 2022). Studies on crops such as wheat, cotton, and tomato also confirm that water-fertilizer integration optimizes plant population structure, enhances resource utilization efficiency, and achieves increased yields and economic benefits (Al-Ghobari et al., 2017; Filintas et al., 2022). However, existing research primarily focuses on the macro-level effects of water-fertilizer integration on yield and resource use efficiency, with insufficient analysis of its underlying mechanisms in regulating crop canopy structure and physiological traits. In particular, systematic studies on canopy uniformity—a core structural characteristic—remain scarce. Most investigations describe canopy changes using single indicators like plant height or leaf area index, failing to reveal the comprehensive regulatory effects of water-fertilizer integration on canopy uniformity. This limitation hinders the optimization and widespread adoption of the technology.

The canopy, as the core site for maize photosynthesis and material production, exhibits structural uniformity (encompassing spatial consistency across structural, coverage, and physiological dimensions) that directly determines the resource utilization efficiency and yield potential of the population (Li et al., 2021; Liu et al., 2021). Therefore, deciphering the regulatory effects of water-fertilizer integration on canopy uniformity is crucial for revealing its yield-enhancing mechanisms. However, traditional canopy uniformity assessments primarily rely on manual point measurements, which present significant limitations and fail to meet the demands of precision agriculture research. Regarding structural assessment, manual measurements of traits like plant height and leaf area typically involve only 30–50 plants per plot (representing approximately 1–2% of total plants). This sample size fails to reflect spatial heterogeneity across the entire plot and introduces substantial measurement errors (e.g., 5–8% error in plant height measurements, and leaf area measurement errors of approximately 10%-12%) (Pérez-Harguindeguy et al., 2013; Araus and Cairns, 2014). Regarding canopy cover assessment, manual estimation relies heavily on visual judgment, exhibiting strong subjectivity with errors reaching 10%-15%. It also fails to quantify minute gaps and continuous cover characteristics (e.g., canopy continuity index, edge smoothness) (Makanza et al., 2018); Regarding physiological assessment, traditional methods predominantly rely on destructive sampling to measure indicators like chlorophyll content and water content. This approach is not only time-consuming and labor-intensive (requiring 2–3 workers for one day per plot) but also incapable of spatiotemporal continuous monitoring. Consequently, it struggles to capture dynamic changes and spatial heterogeneity in canopy physiological states (Cheng et al., 2025). These limitations prevent traditional studies from comprehensively capturing canopy spatial heterogeneity and dynamic changes, let alone achieving multidimensional synergistic quantification of structure-cover-physiology. This severely constrains in-depth analysis of the integrated water-fertilizer regulation mechanism.

The rapid advancement of high-throughput phenotyping technologies has provided revolutionary solutions to overcome traditional research bottlenecks. Among these, unmanned aerial vehicle (UAV)-based multi-source imaging technology has emerged as a core tool for field crop phenotyping due to its unique advantages (Yang et al., 2017; Chivasa et al., 2021). Compared to conventional measurement methods, the application of UAV-based high-throughput phenotyping in this study demonstrates significant synergistic advantages: First, its non-destructive and temporally continuous nature enables repeated monitoring during critical growth stages—such as the jointing stage, tasseling stage, and grain filling stage—without disrupting crop growth, thereby capturing dynamic canopy changes in real time. Research indicates that continuous monitoring throughout the entire growth period can reveal the dynamic evolution of canopy uniformity (e.g., the jointing stage is critical for canopy structural differentiation, while the tasseling stage is central to physiological uniformity formation), whereas traditional single-point measurements only reflect static characteristics at a specific time point (Bendig et al., 2015; Yue et al., 2025). For instance, Bendig et al. (Bareth et al., 2016a) discovered through UAV-based full-growth-stage monitoring that maize canopy height uniformity is most sensitive to water and fertilizer supply during the jointing stage. Water and fertilizer regulation during this critical period can enhance final plant height uniformity by over 20%. Second, high-throughput and large-area coverage capabilities enable simultaneous acquisition of canopy data across multiple maize varieties throughout the entire experimental area in a single pass. This significantly enhances data collection efficiency and effectively addresses the limitation of insufficient sample representativeness in traditional point measurements. For instance, A DJI Mavic 3 Multispectral UAV can complete imaging of a 50-acre test area within one hour, achieving 100% data coverage. In contrast, manual measurement requires 5–8 workers for 1–2 days and yields less than 5% coverage (Sun et al., 2022). Third, the potential for multi-source data fusion. Through the simultaneous acquisition of RGB and multispectral imagery, multiple data types can be concurrently derived, including canopy height models (CHM), orthoimages, and spectral indices (NDVI, RENDVI, WI, etc.). This provides comprehensive support for multidimensional quantification of structural uniformity (e.g., plant height Moran’s I index, texture metrics), coverage uniformity (e.g., gap rate, continuity index), and physiological uniformity (e.g., spectral index coefficient of variation). RGB imagery, with a resolution of up to 2 cm/pixel, enables precise identification of individual canopy profiles and calculation of texture uniformity indices and compactness coefficients. Multispectral imagery can derive vegetation physiological states, quantifying growth vigor, nutrient, and moisture uniformity through indices like NDVI, RENDVI, and WI. Integrating both enables three-dimensional assessment of canopy uniformity (Ma et al., 2023). Fourth, high precision and spatial heterogeneity capture enable pixel-level identification of canopy detail variations, such as local height variability (error ≤3 cm), minute canopy gaps (area ≥0.1 m²), and spectral “hot/cold spots.” This surpasses traditional manual measurements in accurately depicting the spatial distribution characteristics of canopy uniformity. Research confirms that UAV-derived plant height correlates with field measurements at a coefficient exceeding 0.92, while spectral indices correlate with chlorophyll content and water content at coefficients above 0.85 (Bareth et al., 2016). Fifth, quantitative analysis provides a standardized data foundation for objectively calculating canopy uniformity indices, facilitating a shift from qualitative descriptions to quantitative assessments and significantly enhancing the scientific rigor and reliability of research conclusions (Chang et al., 2024). For instance, Moran’s I index calculated using QGIS spatial analysis tools precisely quantifies the spatial autocorrelation of canopy height, providing an objective metric for structural uniformity assessment.

Although existing research has confirmed that precise water and fertilizer management can improve crop canopy growth, current studies still exhibit significant shortcomings: First, most research focuses on analyzing single-dimensional indicators (such as plant height variation and leaf area index), lacking a systematic quantitative and integrated assessment of canopy uniformity across the three dimensions of “structure - coverage - physiology” dimensions of canopy uniformity; second, they fail to fully leverage the advantages of integrating multi-source UAV data, making it difficult to precisely reveal the spatial heterogeneity of canopy uniformity and its intrinsic relationship with yield; third, the differences in responses to integrated water and fertilizer management among various maize varieties remain unclear, limiting the synergistic optimization of technology and varieties and hindering the maximization of the technology’s yield-enhancing potential. These research gaps provide a clear direction for this study.

Based on this, this study utilized 40 maize varieties as experimental materials, establishing water-fertilizer integration (WF) treatments and conventional water-fertilizer management controls (CK). Leveraging multi-source UAV data (RGB + multispectral), a quantitative evaluation system for canopy uniformity was constructed, encompassing structural, coverage, and physiological dimensions. Systematically analyzing the regulatory effects of water-fertilizer integration on maize canopy uniformity, this study clarifies the correlation between key uniformity indicators and yield, revealing the intrinsic regulatory chain: “precision water-fertilizer supply→enhanced canopy uniformity→ efficient resource utilization→increased yield.” It also explores varietal differences in response to water-fertilizer integration and identifies suitable high-yielding varieties. This research aims to provide theoretical support and practical references for optimizing maize water-fertilizer precision management technologies and establishing high-yield cultivation models, while advancing the deep application of UAV-based high-throughput phenotyping technologies in crop physiological and ecological studies.

## 2 Materials and methods

### 2.1 Test site overview

**Figure 1:**
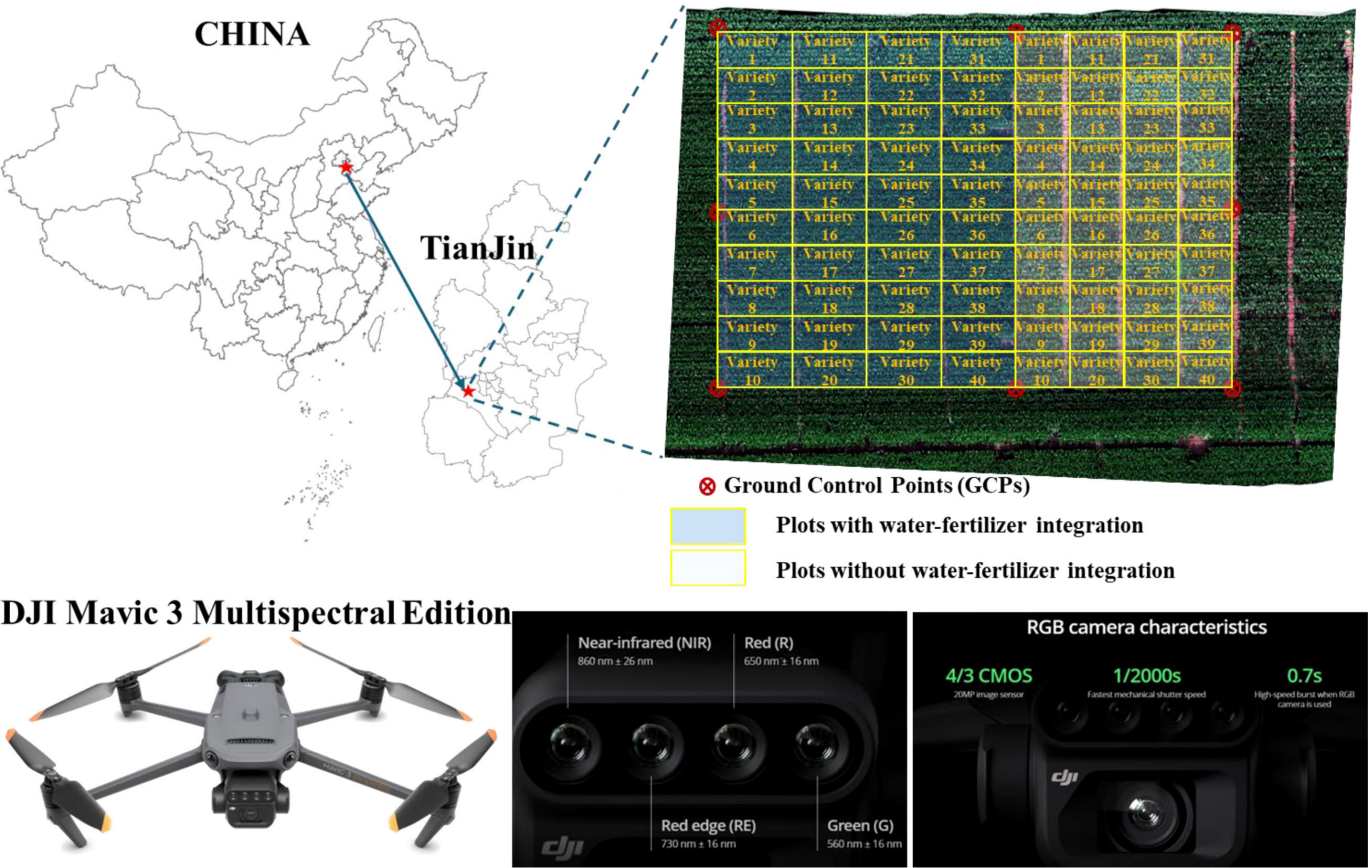
Schematic diagram showing the trial site, data acquisition equipment and planting plan.

The trial was conducted in Tianjin, China in 2024. The trial site is located at the Tianjin Agricultural Experiment Station (specific coordinates omitted), characterized by a warm temperate, semi-humid continental monsoon climate (**Figure 1**). The annual average temperature is approximately 13 °C, with annual precipitation ranging between 550–600 mm, about 70% of which occurs during the summer months of June to August. Annual sunshine hours total approximately 2,400 hours, providing ample light favorable for maize growth. The experimental site features loamy soil (clay loam) with deep, well-drained profiles. Soil organic matter content ranges from 15–20 g/kg, with a pH of approximately 7.5 (slightly alkaline). Available nitrogen, available phosphorus, and available potassium levels in the test soil are approximately 80 mg/kg, 20 mg/kg, and 100 mg/kg, respectively, indicating medium fertility.

### 2.2 Experiment design

#### 2.2.1 Experimental treatments

Two treatments were established: water-fertilizer integration (WF) and conventional water-fertilizer management control (CK). The WF treatment utilized drip irrigation water-fertilizer integration facilities for water and nutrient supply: irrigation water and soluble fertilizers were delivered periodically throughout the maize growth cycle via laid drip irrigation pipes. Water-soluble compound fertilizer (containing balanced nutrients including nitrogen, phosphorus, and potassium) was selected and applied in multiple applications according to maize growth stage requirements. For example, fertilizers were applied with water during the seedling, jointing, tasseling, and grain filling stages, at weekly or decadal intervals. This involved small doses applied at high frequency, with a cumulative nitrogen application rate of approximately N kg/ha (adjusted based on soil fertility). Phosphorus (P_2_O_5_) and potassium (K_2_O) rates were coordinated with nitrogen in a balanced ratio. Under WF treatment, irrigation employs drip irrigation. Water volume and frequency are determined based on soil moisture and maize water requirements, with approximately 8–10 irrigation events throughout the season and a total irrigation volume of 200–300 mm. Ensure each irrigation cycle delivers water uniformly and slowly to allow thorough root zone penetration and consistent supply. Maintain soil relative moisture within the optimal range (e.g., above 70% field capacity) to prevent drought stress and excessive irrigation.

The CK control treatment employed conventional water and fertilizer management practices. Fertilization followed standard soil application methods: a portion of fertilizer (such as compound fertilizer or organic fertilizer) was applied as a basal dressing before sowing, with 1–2 additional manual top-dressings during corn growth (e.g., urea applied during the jointing stage, followed by another nitrogen fertilizer application before tasseling). Fertilizers were uniformly broadcast as granular formulations across the field and then incorporated through irrigation. The total fertilizer application in the CK treatment was equivalent to that in the WF treatment (ensuring consistent nutrient input between the two), but the timing of fertilization was concentrated with longer intervals between applications. Irrigation employed traditional flood irrigation (furrow or ditch irrigation), with a few heavy water applications during critical growth stages (e.g., 3–4 irrigations throughout the entire growth period, each with an irrigation volume of approximately 60–80 mm). Traditional flood irrigation may cause uneven water distribution, potentially leading to waterlogging or insufficient supply in certain field areas. Beyond water and fertilizer supply methods, all other management conditions remained consistent between treatments: corn seeding density, field weeding, pest control, and other management practices were identical to ensure that comparison results stem solely from differences in water and fertilizer management.

#### 2.2.2 Test materials

Forty distinct corn hybrid varieties were used as test materials. Each variety was provided by domestic research institutes or seed companies and is suitable for cultivation in the Tianjin region. These varieties exhibit variations in maturity period and plant architecture (specific variety names omitted). The selection of multiple varieties aimed to evaluate the universality of water-fertilizer integration’s effects on canopy uniformity and yield across corn varieties with different genetic backgrounds.

#### 2.2.3 Plot design

The experiment employed a randomized block design with two replicate blocks. Each replicate contained 80 plots, comprising two water-fertilizer treatments (WF and CK) × 40 variety combinations. The total number of plots across the experiment was 2 × 40 = 80. Each plot measured 30 m² (e.g., 6 m long × 5 m wide). Corn was planted in multiple rows within each plot according to standard corn cultivation practices, ensuring uniform planting density across treatments (approximately 60,000 plants per hectare; row spacing and plant spacing determined based on this density, e.g., 60 cm row spacing and approximately 27 cm plant spacing). Plots of different varieties were randomly arranged within each replicate block to minimize the effects of soil fertility and microtopography variations. Buffer rows or isolation strips were established between adjacent plots, particularly when WF and CK plots were adjacent within the same replicate. This prevented lateral seepage of irrigation water and fertilizers from affecting adjacent flood-irrigated plots by installing impermeable membranes or isolation ditches.

### 2.3 Data acquisition

#### 2.3.1 UAV image acquisition

Maize canopy imagery was captured using the DJI Mavic 3 Multispectral UAV (DJI Mavic 3M). This UAV is equipped with a 20-megapixel visible light RGB camera and four 5-megapixel multispectral cameras, capable of capturing imagery in the green (central wavelength approximately 560 nm), red (650 nm), red edge (730 nm), and near-infrared (860 nm) bands. Prior to aerial photography, flight paths were planned over the field plots, setting the UAV’s altitude to approximately 50 m and flight speed to about 5 m/s. Flight path overlap was configured at 80% fore-aft and 70% sideways overlap to ensure sufficient overlapping areas between adjacent images for subsequent image stitching and 3D reconstruction. The UAV conducted aerial surveys during key growth stages of maize: one flight each at the jointing stage (rapid vegetative growth), tasseling stage (canopy closure), and grain filling stage (seed formation). Surveys were scheduled around midday under clear, windless conditions to ensure uniform lighting and high-quality imagery. Each flight covered the entire experimental area, providing complete imagery for every plot.

The acquired raw imagery undergoes preprocessing before analysis. First, aerial photographs undergo distortion correction and registration using professional image processing software (e.g., DJI Terra, Pix4D, or Agisoft Metashape). Dense point clouds and digital surface models (DSMs) are reconstructed via Structure from Motion (SfM) algorithms. Multiple images are then stitched to generate an Ortho mosaic covering the entire test area, achieving resolutions of approximately 2 cm/pixel (RGB imagery) and 5–8 cm/pixel (multispectral imagery). Building upon the generated DSM, elevation normalization is performed by integrating the trial site’s Digital Elevation Model (DEM) (obtainable through pre-flight aerial photography of bare ground or field measurements) to derive a Canopy Height Model (CHM). Each pixel value in the CHM represents the height of the maize canopy relative to the ground surface at that location. After verifying that the resulting Ortho mosaic and CHM exhibit no significant distortion or mosaic gaps, they are used for subsequent calculations of canopy structure, coverage, and physiological uniformity indices.

#### 2.3.2 Production data acquisition

After maize matures, yield measurements are conducted for each plot. When maize kernels are fully mature and meet harvest standards (typically in late September), each plot is harvested and threshed individually. All corn ears from the plot are collected, and kernels are obtained by threshing. The fresh weight of grains from each plot was measured on-site. A portion of grains was randomly sampled to determine moisture content or dried in an oven to constant weight to calculate moisture percentage. Fresh weights were then converted to dry weights based on a standard moisture content (e.g., 14%). Dry weights were converted to yield per hectare (kg/ha) using the plot area. To ensure measurement accuracy, grains from each plot were strictly segregated during threshing, weighing, and moisture determination to prevent mixing. This yielded grain yield data for 40 varieties under two treatment conditions, enabling analysis of the impact of water-fertilizer integration on yield.

### 2.4 Canopy uniformity index calculation

#### 2.4.1 Structural homogeneity index

##### 2.4.1.1 Canopy height spatial autocorrelation coefficient

Evaluate the spatial autocorrelation of canopy height using acquired CHM data. Calculate Moran’s I index for canopy height pixels in each plot using QGIS spatial analysis tools. Specifically, CHM pixel values within plots serve as observations, with Moran’s I computed based on spatial adjacency matrices. The Moran’s I index typically ranges from -1 to 1. Positive values indicate a positive correlation between adjacent pixel values (i.e., tall clusters with tall, short clusters with short), while negative values indicate a negative correlation (alternating tall and short distributions). In this study, a higher Moran’s I value signifies more uniform spatial distribution of maize plant height, with no pronounced clustering of tall or short plants.

##### 2.4.1.2 Canopy height standard deviation and percentiles

Using Python’s GDAL library to read CHM pixel values, calculate the standard deviation of canopy height values for each plot, along with the 25th and 75th percentile height thresholds. The standard deviation of canopy height reflects the dispersion of plant heights within the entire plot; the 25th and 75th percentiles provide information on the height distribution range (e.g., the 25th percentile corresponds to the height threshold for shorter plants, while the 75th percentile corresponds to the height threshold for taller plants). If the height standard deviation and high quantiles under WF treatment are significantly lower than those in the CK group, it indicates fewer extremely tall and extremely short plants, a more concentrated height distribution, and a larger proportion of medium-height plants. This can be interpreted as a more uniform canopy height structure. Previous studies have employed statistical characteristics such as skewness and kurtosis of plant height histograms to characterize the uniformity of canopy height distribution.

##### 2.4.1.3 Canopy texture uniformity index

Calculates the consistency of canopy texture based on grayscale GLCM (Gray-Level Co-occurrence Matrix) texture analysis of RGB orthoimages. Using ENVI software, texture feature indices—including Energy, Entropy, and Contrast—were extracted from the RGB imagery of each plot. Higher Energy values coupled with lower Entropy and Contrast indicate more uniform image texture and greater canopy structural consistency. In texture analysis, energy reflects the uniformity of gray-level distribution—higher values indicate more uniform texture. Entropy measures uncertainty in gray-level distribution—lower values indicate more ordered texture patterns. Contrast represents the intensity of pixel value variation—lower values indicate smaller gray-level differences between adjacent pixels and smoother texture. Plots with good canopy texture uniformity should exhibit higher GLCM energy and lower entropy and contrast, indicating that the image texture generated by leaf arrangement is more regular and consistent.

##### 2.4.1.4 Crop canopy compactness coefficient

Evaluate the morphological consistency of maize canopies within the same plot using computer vision methods. Employ Python’s OpenCV library to segment individual plants from RGB orthoimages, extracting the projected canopy outline for each maize plant. Subsequently, calculate its compactness. Compactness may be defined as the ratio of a plant’s projected canopy area to its bounding convex hull area, or other metrics reflecting canopy shape compactness (values closer to 1 indicate fuller, smoother canopy contours). The mean and standard deviation of compactness are calculated for all plants within each plot. The coefficient of variation (CV) for compactness is derived as the ratio of standard deviation to mean. A lower compactness CV indicates greater uniformity in canopy shape and size among plants within the plot, reflecting higher structural homogeneity.

#### 2.4.2 Coverage uniformity index

##### 2.4.2.1 Spatial variation coefficient of coverage

Assessing the spatial uniformity of canopy coverage using UAV orthoimages. First, divide the orthoimage of each plot into several equal-area subregions (e.g., 5 m × 5 m grids) based on the grid pattern. Then, perform image binarization (assigning a value of 1 to canopy pixels and 0 to background soil pixels). The proportion of canopy pixels within each sub-area is calculated (i.e., the coverage value for that sub-area). Subsequently, the mean and standard deviation of all sub-area coverage values are determined. The spatial coefficient of variation (CV) for coverage is obtained by dividing the standard deviation by the mean. A lower coverage CV indicates greater similarity in canopy coverage across locations and more uniform spatial distribution; a higher CV suggests greater variation in vegetation coverage between different field locations.

##### 2.4.2.2 Canopy gap rate

Using ENVI software, threshold segmentation is applied to orthoimages to extract non-canopy areas (i.e., bare soil surfaces or vegetation gaps). Based on connectivity analysis, each contiguous non-canopy area is identified and recorded as a “gap.” The number and total area of gaps within each plot were counted. The ratio of total gap area to total plot area was defined as the canopy gap rate. The average gap area was recorded to assist in evaluating gap size characteristics. A lower canopy gap rate indicates more complete maize canopy coverage with minimal large patches of bare ground in the field. Conversely, a higher gap rate suggests uneven plant distribution and the presence of numerous open areas.

##### 2.4.2.3 Coverage continuity index

Calculates the local continuity of canopy coverage using a sliding window method. A 3×3pixel window is slid pixel-by-pixel across the binarized canopy image. For each window, if the central pixel is a canopy pixel, the number of surrounding 8 neighboring pixels belonging to the canopy is counted. This count is then divided by 8 to obtain a local continuity ratio. The average of all continuity ratios across the entire plot is then taken and defined as the Cover Continuity Index. This index ranges from 0 to 1, with values closer to 1 indicating higher connectivity of canopy patches and more continuous vegetation cover. If the Cover Continuity Index of plots treated with WF is higher than that of the CK group, it indicates that field vegetation under drip irrigation and fertilization conditions is more coherent, without fragmented bare patches.

##### 2.4.2.4 Mean edge smoothness of canopy

Edge detection is performed on the binarized canopy coverage area using Python’s Shapely library. Specifically, canopy pixels are clustered into connected regions, and the boundary polygons of each canopy connected region (patch) are extracted. Then, a smoothness metric is calculated for each polygon boundary. For instance, the ratio of the actual boundary perimeter to the perimeter of its corresponding convex hull can serve as a roughness measure (a value closer to 1 indicates smoother, more regular edges). Alternatively, Fourier descriptors can analyze the boundary curve, where the sum of high-frequency components represents irregularity, and 1 minus this irregularity value indicates smoothness. Regardless of the specific algorithm employed, a smoothness score is obtained for each patch. Finally, the average smoothness value across all canopy patches within a subplot is calculated as the mean canopy edge smoothness. A higher mean smoothness indicates overall flatter, less tortuous blocky canopy edge profiles; conversely, a lower mean suggests jagged, highly irregular canopy edges.

#### 2.4.3 Physiological uniformity index

##### 2.4.3.1 Coefficient of variation for NDVI

The Normalized Difference Vegetation Index (NDVI) is calculated from the red and near-infrared bands of multispectral imagery using the formula $NDVI = (NIR - Red)/(NIR + Red)$. For each plot, the NDVI pixel values are collected, and the mean and standard deviation are computed to derive the coefficient of variation (CV) for NDVI. NDVI reflects relative vegetation biomass and growth vigor. A lower CV indicates smaller variations in plant growth within the plot and more uniform canopy biomass distribution. A higher CV suggests that some plants exhibit weaker or stronger growth, resulting in uneven growth within the population.

##### 2.4.3.2 RENDVI spatial variability

Calculate the Red Edge Normalized Difference Vegetation Index (RENDVI) using the red edge band and near-infrared band, following a formula similar to RENDVI = (NIR - RE)/(NIR + RE) * RENDVI correlates closely with chlorophyll content and more sensitively reflects mid-to-late-season nutritional status of vegetation. For each plot’s RENDVI image, calculate the standard deviation and range (maximum minus minimum) of pixel values to quantify spatial variation in RENDVI. Concurrently perform hotspot analysis or other spatial statistics to identify areas with significantly clustered high or low RENDVI values (“hotspots”). If plots treated with WF exhibit lower RENDVI standard deviation and range, with no discernible clusters of high or low values, their canopy nutritional vitality is considered spatially more balanced. Conversely, CK plots may show areas with abnormally low RENDVI (nutrient deficiency) or high RENDVI (excessive growth), resulting in greater spatial heterogeneity.

##### 2.4.3.3 WI coefficient of variation

The Water Index (WI) is calculated to assess the uniformity of canopy moisture conditions. Water indices are typically constructed using near-infrared (NIR) and shortwave infrared (SWIR) bands, such as the Normalized Difference Water Index (NDWI), defined as NDWI = (NIR - SWIR)/(NIR + SWIR). This index correlates with plant canopy moisture content. In this study, WI values were calculated for each plot using near-infrared band data from UAV multispectral imagery and simulated shortwave infrared band data (or calibrated using ground-based leaf water content measurements). The mean and standard deviation of WI pixel values were then determined, with CV representing the spatial variability of WI. A lower CV for WI indicates more consistent plant moisture content across field locations, with no significant local variations in drought or waterlogging. Conversely, a higher CV may suggest insufficient or excessive moisture supply in certain areas, indicating uneven moisture conditions within the population.

##### 2.4.3.4 Standard deviation of estimated chlorophyll content

Use a combination of red edge and green light bands to estimate chlorophyll content in plant leaves and assess its spatial dispersion. Specifically, empirical models proposed in existing research can be adopted, such as using red edge NDVI, green light index, etc., to derive leaf chlorophyll content (relative values) at the individual plant or pixel level. Multispectral pixels within each plot are converted through the model to generate corresponding chlorophyll content estimation images, and then the standard deviation of the pixel values in this image is calculated. A smaller chlorophyll content standard deviation indicates less variation in plant nutrition levels across the field and more uniform nutrient distribution. Conversely, a larger standard deviation suggests that some plants may experience nutrient deficiency or excess, leading to nutritional imbalance within the population. Considering that the model estimates are relative values, we primarily focus on the relative differences in standard deviation between different treatments to evaluate the impact of water and fertilizer management on nutrient uniformity.

### 2.5 Data analysis methods

All experimental data were analyzed using statistical methods. (1) Significance analysis: Independent samples t-tests were conducted on uniformity indices and yield data between the WF group and CK group to determine significant differences between treatments. (2) Correlation analysis: Pearson correlation coefficients were used to assess linear relationships between canopy uniformity indices and maize yield, evaluating each index’s influence on yield. (3) Comprehensive Evaluation: To comprehensively measure canopy uniformity, principal component analysis (PCA) or normalized weighted integrated assessment methods were employed. Multiple uniformity indicators were integrated into a single comprehensive canopy uniformity index to compare the overall uniformity performance across different treatments and varieties; (4) The entire data analysis process was completed using software such as SPSS 26.0, Origin 2022, and Python 3.9. All statistical tests employed a significance level of P < 0.05 as the criterion for determining statistical significance.

## 3 Results and analysis

### 3.1 Effects of water-fertilizer integration on canopy structure uniformity

As shown in **Table 1**, the water-fertilizer integration treatment (WF group) exhibited a more uniform distribution of maize canopy height compared to the conventional treatment (CK group). The spatial autocorrelation coefficient (Moran’s I) for canopy height in the WF group reached 0.78 ± 0.05, significantly higher than the 0.52 ± 0.06 observed in the CK group (P < 0.01). This indicates that adjacent plants exhibited greater height similarity under WF treatment, with tall or short plants no longer showing distinct clustering (i.e., canopy height became more spatially continuous and uniform). Simultaneously, the WF group exhibited lower dispersion in canopy height values, with significantly reduced height standard deviation and upper quantiles compared to the CK group. For instance, both the maximum standard deviation and the 75th percentile of canopy height in the WF group were lower than those in the CK group, indicating that no locally extreme tall or extremely short plant clusters emerged under WF conditions, with height variation effectively suppressed. In other words, water-fertilizer integration helps prevent the formation of distinct “tall-stem” and “short-stem” zones in maize fields, resulting in more uniform plant height across the entire plot.

**Table 1:** Comparison of spatial distribution characteristics of maize canopy height between water-fertilizer integrated management (WF) and conventional management (CK).

Type	Metric	CK	WF	P Value
Canopy height	Spatial autocorrelation coefficient (Moran’s I)	0.52 ± 0.06	0.78 ± 0.05	<0.01
	Standard deviation (cm)	18.6 ± 2.3	9.2 ± 1.5	<0.01
	25th percentile (cm)	152.4 ± 3.1	168.7 ± 2.8	<0.05
	50th percentile (Median, cm)	175.8 ± 3.5	182.3 ± 2.6	<0.05
	75th percentile (cm)	198.5 ± 4.2	190.1 ± 2.9	<0.01
	Maximum (cm)	226.3 ± 5.7	205.8 ± 3.8	<0.01
	Minimum (cm)	128.6 ± 4.8	155.2 ± 3.3	<0.01
	Range (cm)	97.7 ± 6.2	50.6 ± 4.1	<0.01
Canopy texture	Texture energy	18.6 ± 2.5	32.4 ± 3.1	<0.01
	Texture entropy	5.82 ± 0.35	3.67 ± 0.28	<0.01
	Texture contrast	42.3 ± 4.8	21.7 ± 3.2	<0.01
	Canopy compactness per plant (%)	68.5 ± 8.3	75.2 ± 4.1	<0.05
	Coefficient of variation (CV) for canopy compactness (%)	24.6 ± 3.1	12.2 ± 2.3	<0.01

Canopy texture and plant architecture consistency also showed significant improvement under WF treatment. RGB image texture analysis revealed that the WF group exhibited superior canopy texture uniformity compared to the CK group: WF images demonstrated higher texture energy values alongside lower entropy and contrast, indicating more uniform and orderly grayscale distribution in the treated canopy images, with leaves arranged in a more regular texture pattern. In contrast, the CK group exhibited uneven plant growth, resulting in numerous patches of uneven brightness in the image texture. Furthermore, the coefficient of variation (CV) for canopy compactness per plant in the WF group was only about half that of the CK group (CV for plant compactness in WF group significantly lower than CK group, P<0.01). This indicates that under water-fertilizer integration, the size of maize plants and the extent of canopy development were more consistent, leading to better morphological uniformity. Conversely, CK group plants exhibited substantial variation in canopy size and shape, with some displaying lush stems and leaves while others remained relatively short and sparse, resulting in high dispersion of compactness metrics. Overall, water-fertilizer integration enhanced consistency across all aspects of canopy structure through balanced water and nutrient supply. **Table 1** summarizes the comparison results of various canopy structure uniformity indices between the WF and CK groups, revealing that the WF treatment outperformed the CK treatment across all indices.

### 3.2 The effect of water-fertilizer integration on the uniformity of canopy coverage

WF treatment significantly improved the uniformity of canopy coverage within the maize population. Statistics show that the spatial coefficient of variation (CV) of canopy coverage in the WF group was only 8.2% ± 1.3%, significantly lower than the 15.6% ± 2.1% observed in the CK group (P < 0.01) (**Table 2**). This indicates that under WF conditions, vegetation coverage across different areas varied minimally, with no distinct “sparse zones” or “dense zones” observed in the field, resulting in relatively uniform coverage distribution. Conversely, coverage differences were substantial across CK plots, where sparse vegetation and exposed soil patches led to a higher overall coefficient of variation. Additionally, WF treatment reduced canopy gap rates. The WF group exhibited fewer and smaller canopy gaps (continuous areas of exposed soil), with a significantly lower gap rate than the CK group. Within plots of equal size, CK plots often displayed several large patches of bare soil, whereas WF plots showed only scattered, minor gaps. Thus, water-fertilizer integration enabled maize populations to form a denser, more continuous canopy cover, reducing interplant voids.

**Table 2:** Comparison of maize canopy texture and plant architecture consistency indices between water-fertilizer integration treatment (WF) and conventional control treatment (CK).

Metric	CK	WF	P Value
Spatial CV of canopy coverage (%)	15.6 ± 2.1	8.2 ± 1.3	<0.01
Canopy gap rate (%)	9.8 ± 1.7	3.2 ± 0.9	<0.01
Coverage continuity index	0.73 ± 0.06	0.95 ± 0.03	<0.01
Canopy edge smoothness	68.5 ± 4.2	89.3 ± 3.1	<0.01
Mean canopy coverage (%)	82.3 ± 5.1	91.7 ± 3.5	<0.05

**Figure 2:**
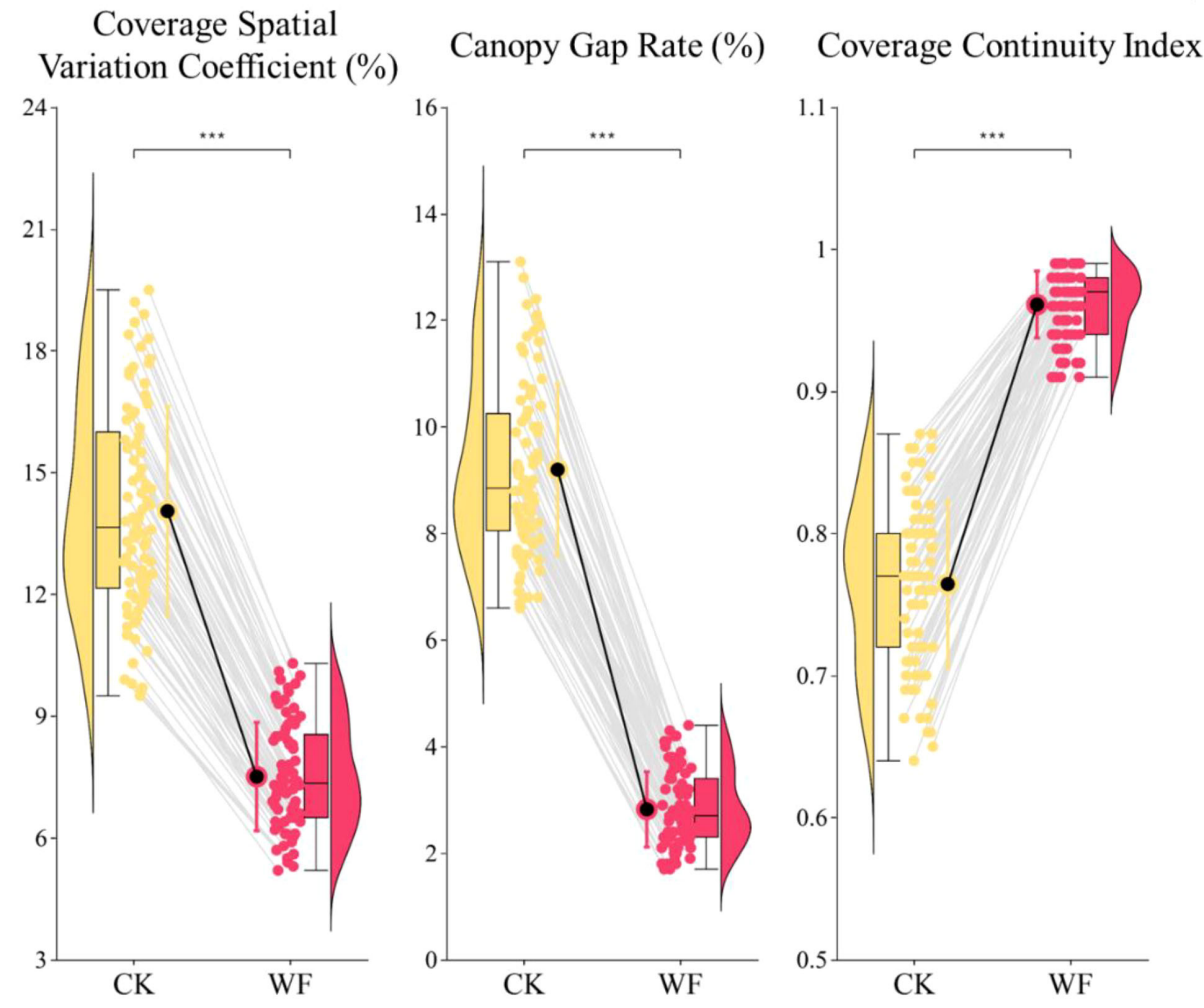
Results of the analysis of the effects of water-fertilizer integration on the uniformity of canopy cover.

**Figure 3:**
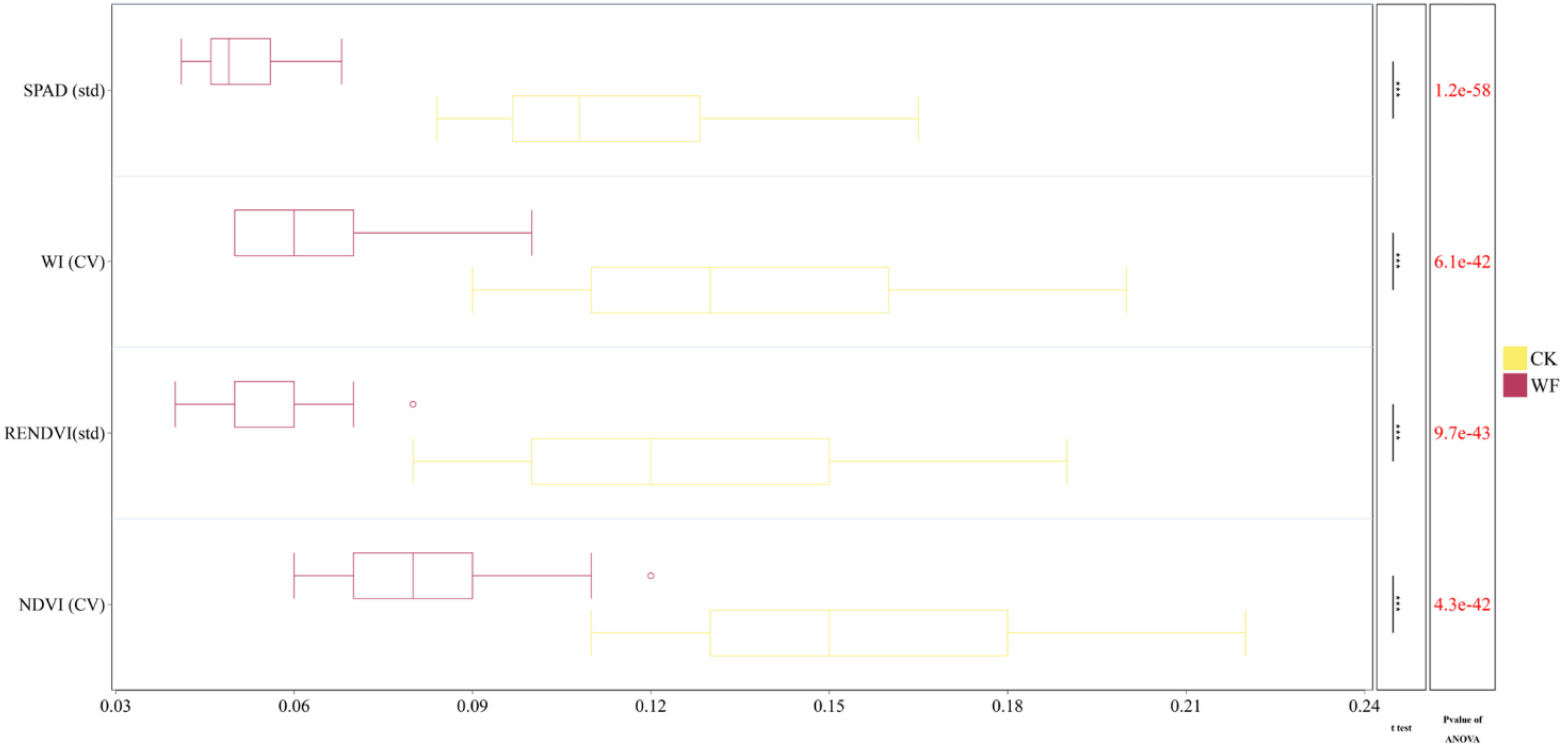
Quantitative analysis of the effects of water-fertilizer integration on the physiological uniformity of the tree canopy.

Canopy cover continuity and edge morphology were also more ideal under WF treatment. The canopy cover continuity index in the WF group approached 1, significantly higher than that in the CK group. This indicates that in plots treated with WF, canopy pixels were largely contiguous, with adjacent pixels predominantly covered by vegetation across most areas, resulting in nearly continuous canopy cover over the entire plot. In contrast, the CK group exhibited a lower canopy cover continuity index, suggesting the presence of numerous fragmented vegetation patches and discontinuous canopy cover. Furthermore, the mean canopy edge smoothness in the WF group was higher than that in the CK group. This means that under WF treatment, the overall canopy contour exhibited smoother and more regular edges without fragmented or jagged boundaries. In contrast, due to uneven plant distribution, the CK treatment showed distinctly uneven and irregular canopy edge shapes. WF treatment demonstrated superior canopy coverage uniformity, both in terms of macro-level continuity and micro-level edge morphology. **Figure 2** compares the spatial coefficient of variation in canopy cover between treatments, revealing that WF treatment substantially reduced spatial variability. **Figure 3** presents a heatmap of canopy gap distribution across the field, visually illustrating significant bare patches in the CK group while the WF group achieved near-continuous canopy coverage.

### 3.3 Effects of water-fertilizer integration on canopy physiological uniformity

Water-fertilizer integration also improved physiological uniformity within the canopy, with spatial consistency of spectral indices consistently superior in the WF group compared to the CK group. First, the coefficient of variation (CV) for NDVI was significantly lower in the WF group than in the CK group. For instance, aerial imagery data during tasseling showed an average NDVI CV of only approximately 0.10 in WF plots, whereas the CK group reached about 0.18 (a difference significant by t-test). This indicates that under balanced water and fertilizer supply conditions via WF, plant growth (reflected by NDVI) was more consistent across the field, without stark contrasts between lushly growing plants and severely malnourished ones. Second, WF treatment reduced spatial heterogeneity in Red Edge NDVI (RENDVI). The standard deviation and range of RENDVI pixel values in the WF plots were both lower than those in the CK plots, and no areas with concentrated distribution of extremely high or low RENDVI values were observed. This indicates that within the WF group, no localized areas exhibited exceptionally vigorous or weakened plants, and chlorophyll content levels across the entire plot remained relatively uniform. In contrast, the CK group frequently displayed “cold spots” (low RENDVI) where plants appeared yellowish and weak, or “hot spots” (high RENDVI) with overly dense, deep-green foliage, reflecting an imbalance in physiological vitality distribution.

The uniformity of canopy water and nutrient distribution was also higher in the WF treatment. The coefficient of variation (CV) results for WI indicates that the CV of the canopy water index in the WF group was significantly lower than that in the CK group. This suggests that uniform water supply via drip irrigation provided similar moisture levels to plants across the canopy, resulting in comparable leaf water status. In contrast, the CK group’s traditional irrigation method often leads to uneven spatiotemporal distribution of soil moisture, with some areas potentially drier and others wetter, causing significant variations in WI values across different field locations. Finally, the standard deviation of estimated chlorophyll content was markedly lower in the WF treatment than in the CK treatment. In other words, under uniform WF fertilization, nutrient uptake was more synchronized and balanced across plants, resulting in similar chlorophyll levels. Conversely, in the CK group, factors like uneven fertilizer distribution or interplant nutrient competition caused substantial variations in chlorophyll content between plants. The aforementioned physiological indicators collectively demonstrate that water-fertilizer integration fosters a more uniform crop canopy. This uniformity is evident across growth vigor (NDVI), nutritional status (RENDVI, chlorophyll), and water status (WI). **Table 3** summarizes the statistical results for canopy physiological uniformity indicators under both treatments, revealing that the WF group exhibited lower variability than the CK group across all metrics.

**Table 3:** Comparison of maize canopy cover characteristics between water-fertilizer integrated management (WF) and conventional management (CK).

Metric	CK	WF	P Value
Spatial CV of canopy coverage (%)	15.6 ± 2.1	8.2 ± 1.3	<0.01
NDVI coefficient of variation (CV)	0.18 ± 0.02	0.10 ± 0.01	<0.01
Red-edge NDVI (RENDVI) standard deviation	0.15 ± 0.02	0.07 ± 0.01	<0.01
Red-edge NDVI (RENDVI) range	0.62 ± 0.05	0.29 ± 0.03	<0.01
Water index (WI) coefficient of variation (CV)	0.16 ± 0.02	0.08 ± 0.01	<0.01
Standard deviation of chlorophyll content estimate (SPAD)	12.8 ± 1.5	5.7 ± 0.8	<0.01
Mean NDVI	0.73 ± 0.04	0.81 ± 0.03	<0.05
Mean red-edge NDVI (RENDVI)	0.78 ± 0.05	0.85 ± 0.03	<0.05
Mean Water index (WI)	0.69 ± 0.04	0.76 ± 0.02	<0.05
Mean chlorophyll content estimate (SPAD)	45.3 ± 3.2	51.6 ± 2.1	<0.05

### 3.4 Correlation analysis between canopy uniformity index and yield

Significant correlations exist between various canopy uniformity indicators and maize yield (**Table 4**). Regarding structural uniformity, the spatial autocorrelation coefficient of canopy height showed a highly significant positive correlation with yield (Pearson correlation coefficient r = 0.82**, P < 0.01), indicating that more uniform distribution of plant height across the field correlates with higher yield. Conversely, the coefficient of variation for individual plant canopy compactness showed a highly significant negative correlation with yield (r = -0.76**, P < 0.01), meaning that greater morphological variation among plants (inconsistent plant types) resulted in lower yields. This demonstrates that the uniformity of plant height and morphological consistency within a maize population significantly influence final yield: populations with more uniform structures tend to achieve higher yields.

**Table 4:** Comparison of physiological uniformity indices in maize canopies between water-fertilizer integrated management (WF) and conventional management (CK).

Metric	CK	WF	P Value
Spatial coefficient of variation for canopy coverage (%)	15.6 ± 2.1	8.2 ± 1.3	<0.01
NDVI coefficient of variation (CV)	0.18 ± 0.02	0.10 ± 0.01	<0.01
Red-edge NDVI (RENDVI) standard deviation	0.15 ± 0.02	0.07 ± 0.01	<0.01
Red-edge NDVI (RENDVI) range	0.62 ± 0.05	0.29 ± 0.03	<0.01
Water index (WI) coefficient of variation (CV)	0.16 ± 0.02	0.08 ± 0.01	<0.01
Standard deviation of estimated chlorophyll content (SPAD)	12.8 ± 1.5	5.7 ± 0.8	<0.01
Mean NDVI	0.73 ± 0.04	0.81 ± 0.03	<0.05
Mean red-edge NDVI (RENDVI)	0.78 ± 0.05	0.85 ± 0.03	<0.05
Mean water index (WI)	0.69 ± 0.04	0.76 ± 0.02	<0.05
Mean estimated chlorophyll content (SPAD)	45.3 ± 3.2	51.6 ± 2.1	<0.05

The correlation between vegetation coverage uniformity and yield is equally pronounced. The spatial variation coefficient of canopy cover showed a highly significant negative correlation with yield (r = -0.79**, P<0.01), indicating that greater variation in vegetation cover within plots (i.e., distinct gaps and dense patches) was associated with lower overall yield. Conversely, the canopy continuity index exhibited a highly significant positive correlation with yield (r = 0.75**, P<0.01). In other words, plots with higher canopy connectivity and less bare ground tended to yield higher. These two indices illustrate the significance of canopy cover consistency for yield formation from both positive and negative perspectives: a uniform and continuous canopy can more effectively intercept and utilize light energy, leading to higher photosynthetic efficiency within the community and consequently higher yields.

Regarding physiological uniformity indicators, the NDVI coefficient of variation showed a highly significant negative correlation with yield (r = -0.80**, P<0.01). This indicates that more uneven distribution of plant growth leads to greater yield losses; conversely, higher NDVI uniformity signifies that all plants achieve similar growth levels, resulting in higher group yields. Similarly, the coefficient of variation for the canopy water index (WI) showed a highly significant negative correlation with yield (r = -0.73**, P<0.01). This indicates that balanced field moisture supply influences yield, with high-yielding plots typically exhibiting consistent plant water status across all locations, free from weak plants caused by localized drought or waterlogging. Therefore, whether assessed from vegetation vitality (NDVI) or water status (WI), physiological uniformity contributes to high yields. Further analysis revealed that combining multiple uniformity metrics into a composite canopy uniformity index yielded a more pronounced correlation with yield. There is a clear positive correlation between the composite canopy uniformity score and yield across plots, indicating higher yields in more uniform areas. This collectively validates the logic: “integrated water and fertilizer management→enhanced canopy uniformity→increased yield.” **Table 3** details the Pearson correlation coefficients between major uniformity indicators and yield, with coefficients marked “**” being statistically significant at the 1% level.

### 3.5 Differences in response to fertigation among varieties with varying canopy uniformity

Different maize varieties exhibit significant variations in the extent of canopy uniformity improvement under water-fertilizer integration conditions. Overall, the majority of varieties showed enhanced uniformity indices and corresponding yield increases under WF treatment, though the magnitude of improvement varied by variety. Some varieties responded strongly to water-fertilizer integration. For example, a high-yielding variety A demonstrated a comprehensive canopy uniformity index approximately 35% higher than the CK group under WF conditions, with a corresponding 28% yield increase; while another variety B showed only a 10% improvement in uniformity index and less than a 5% yield increase (**Table 5**). Based on these results, we identified a group of varieties highly responsive to WF. These varieties exhibited more consistent growth and significant yield gains under precise water and fertilizer supply. Conversely, a few varieties responded weakly to WF, with uniformity indices and yields showing little difference from the CK treatment.

**Table 5:** Results of pearson correlation analysis between maize canopy uniformity indicators and yield.

Category	Metric	R	P Value	Mark
Structural uniformity	Canopy height spatial autocorrelation coefficient	0.82	<0.01	**
	Coefficient of variation for canopy compactness (%)	-0.76	<0.01	**
Coverage uniformity	Spatial coefficient of variation for canopy coverage (%)	-0.79	<0.01	**
	Coverage continuity index	0.75	<0.01	**
Physiological uniformity	NDVI coefficient of variation (CV)	-0.80	<0.01	**
	Red-edge NDVI (RENDVI) standard deviation	-0.74	<0.01	**
	Water index (WI) coefficient of variation (CV)	-0.73	<0.01	**
	Standard deviation of estimated chlorophyll content (SPAD)	-0.71	<0.01	**
Comprehensive index	Integrated canopy uniformity index	0.88	<0.01	**

**Figure 4:**
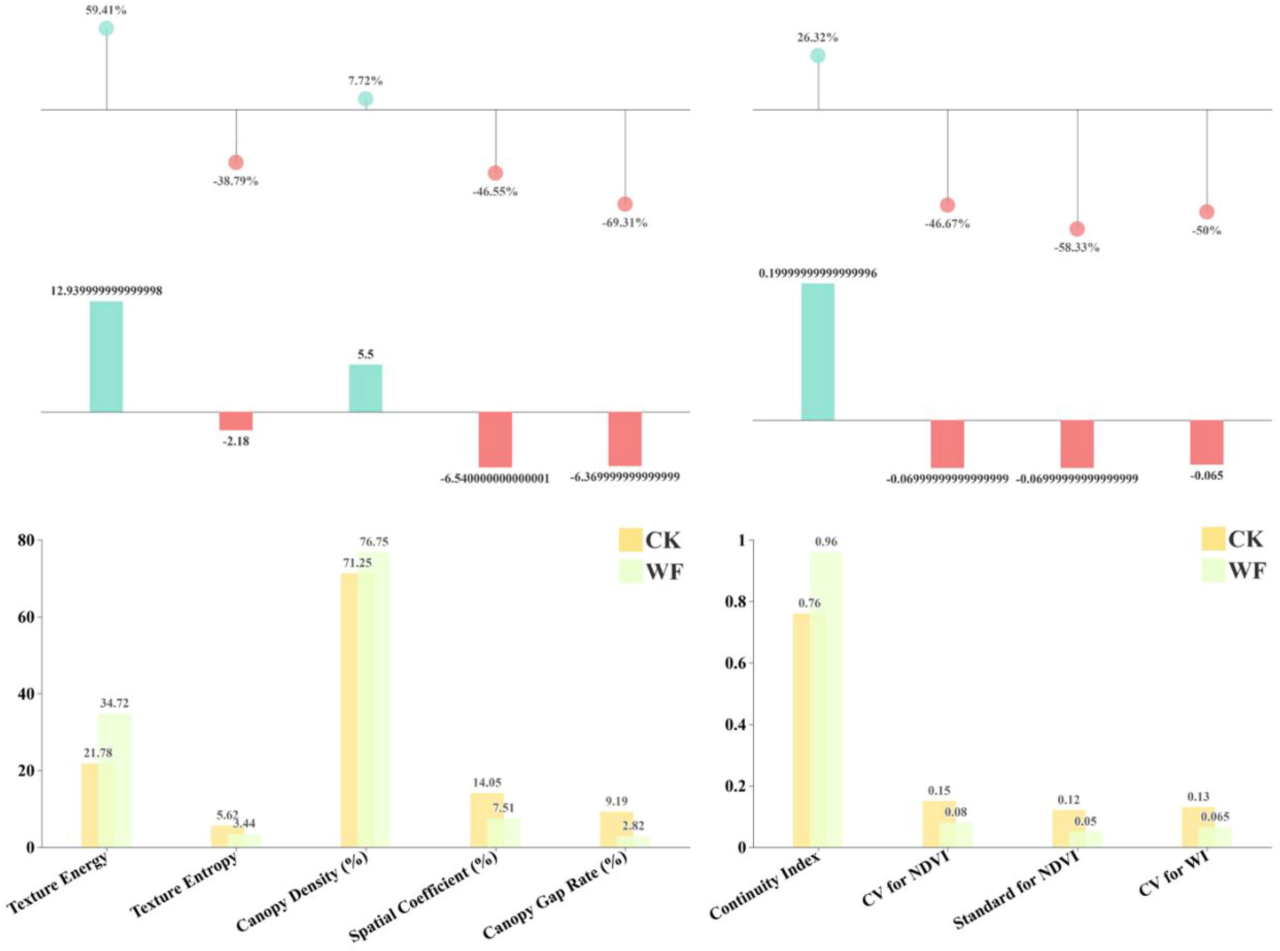
Differences in the percentage increase in the comprehensive canopy uniformity index versus the percentage increase in yield across 40 varieties under integrated water and fertilizer management conditions.

At the variety level, improvements in canopy uniformity typically correlate positively with yield increases. **Figure 4** compares the percentage increase in canopy uniformity composite index versus the percentage yield increase for 40 varieties under WF treatment. Most data points cluster along a positive correlation trendline: varieties showing more pronounced uniformity improvements also exhibit greater yield gains. Of course, some variation exists among different maize varieties. A few varieties showed improved uniformity without a significant yield increase, indicating that yield is also constrained by factors beyond uniformity. Overall, however, this result provides a basis for screening and promoting maize varieties suitable for water-fertilizer integration cultivation. In future production, priority should be given to varieties that can fully realize their yield potential under uniform water and fertilizer supply conditions, thereby maximizing the yield-enhancing effects of water-fertilizer integration technology.

## 4 Discussion

### 4.1 Regulatory mechanisms of water-fertilizer integration in enhancing canopy uniformity

The reason water-fertilizer integration enhances canopy uniformity can be explained through the lens of water and nutrient supply patterns. Traditional flood irrigation and single-application fertilization often result in uneven distribution of water and nutrients across fields: soil moisture near the water inlet is high while distant areas remain low. After heavy irrigation, soil moisture rapidly percolates downward, potentially causing drought stress in some areas while root systems suffer from oxygen deprivation due to waterlogging in others. Single-application fertilization with large quantities of nutrients can easily cause nutrient excess (or even leaching loss) in some areas, while other areas suffer from insufficient nutrient supply due to inadequate diffusion. Under this supply method, plants in different locations face vastly different environmental conditions, inevitably leading to uneven growth. In contrast, water-fertilizer integration employs drip irrigation systems with buried pipes to deliver water and nutrients “on schedule and in precise quantities” uniformly to the root zones of each plant. This ensures consistent soil moisture and nutrient conditions across the entire field. Drip irrigation provides slow, localized watering, unlike flood irrigation where water flows across the surface. Consequently, soil moisture remains spatially balanced and at optimal levels. Soluble fertilizers applied with water permeate uniformly into the root zone soil, effectively preventing localized nutrient accumulation or deficiency while reducing growth disparities caused by water stress. Under this balanced water and nutrient supply, maize plants’ roots distribute more evenly throughout the tillage layer rather than clustering in localized “nutrient pockets.” This balanced root expansion provides stable and consistent resource supply for the aboveground canopy, enabling more uniform canopy development: differences in plant height and individual biomass decrease, and the overall growth of the population tends toward uniformity. Existing research indicates that precise supply measures, such as alternating irrigation combined with uniform nitrogen application, can significantly increase the Leaf Area Index (LAI) during the later growth stages of maize, thereby promoting yield formation.

Furthermore, improved structural uniformity positively impacts the microclimate and light conditions within the canopy. A highly uniform canopy implies minimal height variation between plants and low stratification, resulting in more equitable light distribution within the stand. Without tall plants severely shading shorter ones, each plant receives comparable light exposure, ensuring equal opportunities for photosynthesis. Consequently, all leaves participate in effective photosynthesis. This prevents situations where upper leaves of some plants become excessively shaded while lower leaves of shorter plants remain chronically deprived of light, thereby reducing wasteful light interception. Furthermore, standardized spacing between plants and rows reduces excessive competition pressure. The uniform spatial pattern ensures that each maize plant has ample and comparable growing space and resource access, preventing local overcrowding or sparse conditions from limiting individual growth potential. Simultaneously, the uniform canopy structure lays the foundation for physiological uniformity. Consistent plant height and leaf area mean each plant experiences a similar microclimate (with comparable light, humidity, and ventilation), leading to more synchronized transpiration, photosynthesis, and nutrient uptake. No plant is “late blooming” due to insufficient light or poor ventilation, nor is any severely lagging from overexposure or lack of water and nutrients. When all plants grow under comparable conditions, the number of disadvantaged individuals within the population is significantly reduced. Each plant can fully realize its productive potential, leading the entire group to achieve a synergistic and efficient state. In summary, water-fertilizer integration enhances the coordinated uniformity of canopy structure and physiology by enabling “uniform underground resource supply→uniform above-ground plant growth.” This regulatory mechanism can be summarized as a chain process: “uniform water and fertilizer supply→uniform root growth→ uniform canopy structure→uniform canopy physiology.” In this study, we observed that the WF treatment outperformed the CK treatment in reducing plant height variation and decreasing canopy spectral index variability, validating each link in the above mechanism: precise water and fertilizer management comprehensively enhances canopy uniformity.

### 4.2 The mechanism linking canopy uniformity to yield

The results of this study clearly demonstrate a close intrinsic relationship between enhanced canopy uniformity and increased yield. This relationship can be explained from the perspective of resource use efficiency within crop populations.

First, improved structural uniformity implies greater consistency in morphological parameters such as plant height and leaf area. This reduces the inefficiency in light utilization caused by uneven plant heights. When plants vary significantly in height, taller plants often shade shorter ones, hindering the latter’s ability to perform photosynthesis efficiently. simultaneously, leaves at the tops of tall plants may reach light saturation, effectively wasting the excess sunlight they capture. However, when plants are of similar height, the entire canopy functions as a flat “light-receiving surface.” Sunlight can then uniformly reach every leaf, providing each plant with nearly equal access to light, thereby significantly enhancing the group’s overall light utilization efficiency. Moreover, uniform and reasonable planting density and row spacing reduce unnecessary competition between plants. An optimized spatial layout ensures that each maize plant has sufficient and equal access to growing space, nutrients, and water supply. This prevents stunted growth due to crowding or land waste from excessive spacing. Thus, structural uniformity minimizes internal competition within the group, maximizes synergistic effects, and fully unleashes the yield potential of each individual plant.

Second, improved canopy uniformity reduces the proportion of “photosynthetic gaps” in the field. These gaps—areas without crops—allow direct sunlight to reach the soil surface without absorption. This not only wastes solar energy but also accelerates soil moisture evaporation and nutrient leaching due to lack of plant uptake. Extensive research and practical experience confirm that even scattered small gaps negatively impact total yield. For instance, one study found that when maize rows contained extended gaps between plants (approximately 1.25–2 m), yields decreased by about 5–6% compared to ideal stands without gaps. Therefore, if numerous or large canopy gaps exist in the field, yield per unit area will inevitably be significantly affected. Conversely, when canopy coverage achieves high uniformity and continuity, nearly every square meter of the field has leaves performing photosynthesis. The total leaf area of the stand is fully utilized, with yield contributed by the entire land area without dead zones.

Finally, physiological uniformity ensures that all plants within the stand perform photosynthesis and grow with similar efficiency. If some plants within the population exhibit significantly weaker growth due to water or nutrient deficiencies, they not only contribute lower yields themselves but also consume resources while producing less dry matter—effectively reducing resource utilization efficiency. Conversely, when all plants maintain good nutritional and hydrological conditions with minimal variation, each plant efficiently converts invested resources into grain yield, eliminating any “lagging” weak plants. When indicators like NDVI, chlorophyll content, and water index (WI) are highly consistent across plants, it indicates no severe nutrient deficiencies or excesses within the population. Photosynthetic and transpiration rates are comparable across leaf areas, leading to balanced dry matter accumulation. Consequently, overall yield is no longer constrained by a few weak plants, significantly boosting the collective output.

In summary, uniformity across the three dimensions—canopy structure, coverage, and physiology—mutually reinforces each other, collectively influencing final yield formation. Structural uniformity provides an optimal framework for light energy capture; uniform coverage ensures sufficient effective leaf area participates in photosynthesis; and physiological uniformity guarantees all plants operate at peak efficiency. Integrated water and fertilizer management precisely achieves this by simultaneously enhancing the uniformity of these “three pillars.” This significantly boosts the crop stand’s overall efficiency in utilizing resources like light, water, and nutrients, ultimately leading to a marked increase in yield. This process can be summarized as: “Integrated water and fertilizer management → Enhanced canopy uniformity → Improved resource utilization efficiency of the crop stand → Increased yield.” This sequence aligns closely with the measured data and correlation analysis results from this study.

### 4.3 Comparison of this study with existing research

Compared to existing research, our work demonstrates innovation and depth in multiple aspects. First, we established a three-dimensional canopy uniformity evaluation system encompassing “structure-cover-physiology.” Previous studies often described crop stand growth uniformity at a qualitative level or relied solely on single indicators (such as coefficient of variation in plant height or leaf area distribution within stands) for assessment. While some studies noted that precision management practices like drip irrigation can promote more uniform crop growth, precise quantitative metrics to characterize this phenomenon were lacking. This study integrates multi-source remote sensing and image processing techniques to design a multi-indicator synergistic assessment method targeting different aspects of canopy uniformity. For instance, we simultaneously considered spatial patterns of canopy height (Moran’s I, etc.), height distribution dispersion (standard deviation, quantiles), image texture uniformity (GLCM energy, entropy, contrast), and vegetation index variability (CV of NDVI, RENDVI, WI). This approach not only quantifies the impact of water-fertilizer integration on canopy uniformity but also identifies which aspects show the most significant improvement. This addresses previous gaps in quantifying canopy uniformity, providing a more comprehensive and in-depth understanding of population uniformity.

Second, we delved into the relationship between canopy uniformity and yield, clearly identifying key uniformity indicators closely correlated with yield. While many existing studies report the extent of water and fertilizer management effects on yield (e.g., drip irrigation yielding more than flood irrigation) or vaguely mention the positive impact of crop population growth consistency on yield, few provide quantitative correlation analyses. This study quantitatively links variations in canopy uniformity to yield changes using methods like Pearson correlation analysis. For instance, we found that spatial autocorrelation coefficients for canopy height, spatial variation coefficients for canopy cover, and NDVI variation coefficients are core indicators reflecting the relationship between canopy uniformity and yield. Their correlation coefficients with yield ranged from 0.75 to 0.82 (absolute values), demonstrating high statistical significance. These findings not only validate the hypothesis that canopy uniformity influences yield but also identify key remote sensing indicators for rapid field diagnostics. This represents a gap in previous literature, thereby enriching our understanding of the yield-enhancing mechanisms of precision water and fertilizer management.

Moreover, unlike previous studies that often focused on single indicators, our multidimensional integrated analysis strategy reveals the comprehensive impact of integrated water and fertilizer management on crop populations. Early research may have concentrated solely on drip irrigation’s effects on plant height or biomass variability, or solely on changes in spectral indicators like NDVI. Our findings demonstrate that structural, canopy cover, and spectral aspects are actually interconnected and change collectively. Through multidimensional indicator combinations, we identified correlations and synergistic interactions among these changes. For instance, we observed that enhanced structural uniformity often coincided with improved physiological uniformity, with both factors collectively driving yield increases. This understanding of synergistic effects contributes to a comprehensive grasp of water-fertilizer integration’s operational mechanisms.

In summary, this study innovatively establishes a multi-indicator quantitative assessment system for canopy uniformity. It quantitatively reveals the intrinsic mechanisms by which water-fertilizer integration enhances canopy uniformity and its relationship with yield. Compared to previous fragmented qualitative conclusions, our research provides stronger data support for precision water-fertilizer management to boost maize yields. It also offers a reference indicator system and analytical approach for subsequent related studies.

### 4.4 Research limitations and future directions

Although this study yielded relatively systematic results, several limitations exist that warrant improvement in future work. First, our data collection focused on several key growth stages of maize, without continuous monitoring of canopy uniformity changes throughout the entire growth period. Canopy uniformity may exhibit dynamic variations during development—for example, uneven growth among seedlings in the early stages, gradual uniformity after the jointing stage, and potential re-emergence of unevenness in the late grain-filling stage due to premature senescence or lodging in some plants. This dynamic information was not captured in this study. Future research could enhance temporal resolution by increasing the frequency of aerial monitoring to track changes in canopy uniformity throughout the growth cycle. This would help identify which stage’s uniformity most significantly impacts final yield, thereby optimizing intervention timing.

This experiment utilized only 40 maize hybrid varieties, representing relatively limited diversity in both number and genetic backgrounds. Different maize genotypes may respond variably to optimized water and fertilizer management. Patterns observed in a small number of varieties may not fully apply to all maize types. Future studies should expand the range of tested varieties to include more maize types with different maturity periods and plant architectures. This will validate whether the relationship between enhanced canopy uniformity and increased yield holds across a broader genetic background and identify the variety types that respond best to integrated water and fertilizer management. This holds practical significance for promoting water-fertilizer integration technology and variety combinations tailored to local conditions across regions. Technologically, future approaches could integrate novel remote sensing and sensing technologies to broaden the dimensions of canopy uniformity assessment. Hyperspectral remote sensing captures finer spectral information of crop canopies, enabling the development of indicators such as canopy nitrogen content uniformity and photosynthetic potential uniformity using hyperspectral imagery. Thermal infrared remote sensing can monitor canopy surface temperature distribution to evaluate canopy temperature uniformity (closely related to plant transpiration rates and water use efficiency). Incorporating these new indicators into the uniformity evaluation system can more comprehensively reflect the impact of water and fertilizer management measures on the uniformity of physiological and ecological processes within crop populations. Additionally, deploying IoT soil moisture sensors and stem flow sensors in the field could enable real-time monitoring of soil water and nutrient distribution alongside plant physiological status, providing more direct data support for understanding uniformity formation. Furthermore, there is room for further research in experimental design. This study primarily compared two modes— “integrated water and fertilizer management vs. conventional management”—with binary water and fertilizer input levels. Future studies could employ gradient experiments (e.g., varying total irrigation volumes, total fertilizer application rates, or fertilization frequencies) to investigate how canopy uniformity changes with water and fertilizer management intensity. This would identify the minimum inputs required for significant uniformity improvement and the threshold beyond which uniformity no longer increases markedly, guiding optimized water and fertilizer inputs in practical production. In other words, determining “sufficient versus excessive water and fertilizer inputs” is crucial to maximize water and fertilizer use efficiency while ensuring uniformity and yield.

Finally, from the perspectives of crop genetic improvement and physiological mechanisms, deeper research can be conducted on the differential responses of canopy uniformity among different varieties. We observed that some varieties showed significant improvements in both yield and uniformity under optimized water and fertilizer conditions, while others exhibited relatively modest gains. This suggests potential differences among varieties in root architecture, nutrient uptake and distribution, and interplant competition capabilities. Future work could involve comparative analysis of physiological and biochemical indicators between high- and low-response varieties, extending to gene expression profiling and genome-wide association studies. This would identify key genes or regulatory networks governing plant growth consistency and balanced resource utilization. Such insights would facilitate breeding new crop varieties highly adaptable to precision water and fertilizer management, characterized by high uniformity within populations, thereby achieving synergistic optimization of cultivation practices and varietal traits.

In summary, further research should expand data acquisition both “horizontally”—by broadening the spatiotemporal scope and variety diversity—and “vertically”—by delving deeper into the physiological and molecular mechanisms through which canopy uniformity influences yield. This knowledge should then be applied to enhance agronomic management and breeding practices. As research progresses, precision agricultural technologies like integrated water and fertilizer management are expected to play an increasingly significant role in ensuring increased grain production and efficiency.

## 5 Conclusion

This study utilized 40 maize varieties, comparing water-fertilizer integration (WF) treatments with conventional water-fertilizer management controls (CK). Leveraging UAV-derived RGB + multispectral data, a three-dimensional uniformity evaluation system was established to quantify canopy “structure-cover-physiology.” The research systematically investigated its regulatory effects and mechanisms. Results indicate that water-fertilizer integration significantly enhanced three-dimensional uniformity in maize canopy structure (increased spatial autocorrelation coefficient for plant height, reduced height standard deviation, more regular texture, and more uniform plant architecture), coverage (decreased spatial coefficient of variation for coverage and gap rate, improved continuity and edge smoothness), and physiology (reduced coefficients of variation for NDVI, RENDVI, WI, and other indicators, along with decreased standard deviation of estimated chlorophyll content). Canopy uniformity indices showed extremely significant correlations with yield, with spatial autocorrelation coefficient of canopy height, spatial coefficient of variation of canopy cover, and NDVI coefficient of variation serving as core correlation indicators. The comprehensive index of canopy uniformity correlated with yield at a coefficient of 0.88 (P<0.01). Different maize varieties exhibited significant variations in response to water-fertilizer integration. Some varieties showed over 30% improvement in the comprehensive uniformity index and 28% yield increase, while a few varieties responded weakly; Integrated water and fertilizer management enhances yield through a chain mechanism: “uniform water and fertilizer supply → uniform root growth → uniform three-dimensional canopy → efficient utilization of population resources → increased yield.” These findings provide theoretical support and practical guidance for precise water and fertilizer management in maize, the development of high-yield cultivation models, and the selection of suitable varieties.

## Data Availability

The original contributions presented in the study are included in the article/**Supplementary Material**. Further inquiries can be directed to the corresponding author.

## Supplementary material

Supplementary Table 1 Comprehensive water and fertilizer management schedule.

Supplementary Table 2 The main varieties with their key agronomic traits.
