# Supplementary material for: The regulatory effects of water-fertilizer integration on maize canopy uniformity and yield: a comprehensive evaluation based on multi-source UAV data
Source: Front Plant Sci. 2026 Jul 1;17:1778586. doi: 10.3389/fpls.2026.1778586 (PMC13369051; doi:10.3389/fpls.2026.1778586)
Supplement: Supplementary Table 1 — Comprehensive water and fertilizer management schedule. [file Table1.docx]

**Supplementary Table S1.** Comprehensive water and fertilizer management schedule.

| **Growth Stage** | **Days After Sowing** | **N (kg/ha)** | **P₂O₅ (kg/ha)** | **K₂O (kg/ha)** | **Irrigation (mm)** | **CK**  **N (kg/ha)** | **CK**  **Irrigation (mm)** |
| --- | --- | --- | --- | --- | --- | --- | --- |
| Seedling | 0-30 | 45 | 30 | 30 | 25 | 90 (basal) | 70 |
| Jointing | 31-55 | 60 | 20 | 25 | 30 | 60 (topdress 1) | 75 |
| Tasseling | 56-75 | 60 | 15 | 30 | 35 | - | 80 |
| Grain Filling | 76-100 | 35 | 10 | 15 | 25 | - | 75 |
| Total | - | 200 | 75 | 100 | 115 | 150 | 300 |

**Supplementary Table S2.** The main varieties with their key agronomic traits.

| **No.** | **Variety Name** | **Breeding Company** | **Maturity Group** | **Plant Type** | **Plant Height (cm)** | **Ear Height (cm)** |
| --- | --- | --- | --- | --- | --- | --- |
| 1 | ZD 958 | Henan Academy | Medium (125d) | Compact | 265±8 | 115±5 |
| 2 | XY 335 | Sinochem | Medium (128d) | Semi-compact | 278±10 | 120±6 |
| 3 | JK 968 | Beijing Academy | Medium (126d) | Compact | 270±9 | 118±5 |
| ... | ... | ... | ... | ... | ... | ... |
| 40 | DK 517 | Dika Seeds | Early (118d) | Spreading | 245±7 | 105±4 |
